# Molecular epidemiology of multidrug-resistant *Klebsiella pneumoniae, Enterobacter cloacae*, and *Escherichia coli* outbreak among neonates in Tembisa hospital, South Africa

**DOI:** 10.3389/fcimb.2024.1328123

**Published:** 2024-02-28

**Authors:** John Osei Sekyere, Masego Mmatli, Anel Bosch, Ramathetje Virginia Ntsoane, Harishia Naidoo, Sinenhlanhla Doyisa, Nontuthuko E. Maningi, Nontombi Marylucy Mbelle, Mohamed Said

**Affiliations:** ^1^ Department of Medical Microbiology, School of Medicine, University of Pretoria, Pretoria, South Africa; ^2^ Department of Dermatology, School of Medicine, University of Pretoria, Pretoria, South Africa; ^3^ Institute of Biomarker Research, Medical Diagnostic Laboratories, Genesis Biotechnology Group, Hamilton, NJ, United States; ^4^ National Health Laboratory Services, Tshwane Academic Division (TAD), Pretoria, South Africa; ^5^ Department of Microbiology, School of Life Sciences, University of KwaZulu-Natal, Durban, South Africa

**Keywords:** carbapenem, neonates, sepsis, outbreak, infection control, colistin, drug resistance, Pretoria

## Abstract

**Background:**

An outbreak of multidrug-resistant *Klebsiella pneumoniae, Escherichia coli, and Enterobacter cloacae* infections in a neonatal ward within a tertiary hospital in South Africa resulted in the mortality of 10 patients within six months. In this work, the genomic epidemiology of and the molecular factors mediating this outbreak were investigated.

**Methods:**

Bacterial cultures obtained from clinical samples collected from the infected neonates underwent phenotypic and molecular analyses to determine their species, sensitivity to antibiotics, production of carbapenemases, complete resistance genes profile, clonality, epidemiology, and evolutionary relationships. Mobile genetic elements flanking the resistance genes and facilitating their spread were also characterized.

**Results:**

The outbreak was centered in two major wards and affected mainly neonates between September 2019 and March 2020. Most isolates (n = 27 isolates) were *K. pneumoniae* while both *E. coli* and *E. cloacae* had three isolates each. Notably, 33/34 isolates were multidrug resistant (MDR), with 30 being resistant to at least four drug classes. All the isolates were carbapenemase-positive, but four *bla*_OXA-48_ isolates were susceptible to carbapenems. *Bla*_NDM-1_ (n = 13) and *bla*_OXA-48/181_ (n = 15) were respectively found on *IS*91 and *IS*6-like *IS*26 composite transposons in the isolates alongside several other resistance genes. The repertoire of resistance and virulence genes, insertion sequences, and plasmid replicon types in the strains explains their virulence, resistance, and quick dissemination among the neonates.

**Conclusions:**

The outbreak of fatal MDR infections in the neonatal wards were mediated by clonal (vertical) and horizontal (plasmid-mediated) spread of resistant and virulent strains (and genes) that have been also circulating locally and globally.

## Introduction

*Escherichia coli, Enterobacter cloacae* complex, and *Klebsiella pneumoniae* are three of the most challenging Gram-negative bacterial pathogens implicated in numerous nosocomial infections and outbreaks (Spyropoulou et al., 2016; Osei Sekyere and Reta, 2020a; Osei Sekyere et al., 2021). They are mostly associated with multidrug resistance in urinary tract infections, bacteremia, meningitis, and pneumonia (Osei Sekyere and Reta, 2020a; Osei Sekyere and Reta, 2020b; Perovic et al., 2020). Among infants, these two pathogens are mainly associated with blood-stream infections (sepsis) and pneumonia. Outbreaks among neonatal units and these bacteria are usually antibiotic resistant (Osei Sekyere et al., 2021).

*E. coli, E. cloacae*, and *K. pneumoniae* have been associated with resistance to carbapenems, colistin, tigecycline, other β-lactams, fluoroquinolones, aminoglycosides, tetracyclines, fosfomycin, and all other antibiotic classes (Jean et al., 2015; Osei Sekyere and Reta, 2020a; Osei Sekyere and Reta, 2020b; Ramaloko and Osei Sekyere, 2022). It is therefore not uncommon for these species to harbor several resistance determinants and express multidrug resistance phenotypes. Furthermore, highly virulent strains, including hypervirulent *K. pneumoniae*, have emerged and several reports have shown the presence of multidrug resistance and hypervirulence in nosocomial and community *K. pneumoniae* strains (Fu et al., 2018; Shu et al., 2019).

From September 2019, physicians at Tembisa hospital, South Africa, began to see a spike in neonatal infections. The pediatrician notified the Department of Medical Microbiology at the University of Pretoria and requested an investigation into the cause of the spike in infection among neonates. Unfortunately, while preparations were ongoing to initiate the investigations, an outbreak occurred between November 2019 and January 2020 (Essel et al., 2020). Twenty infants were infected with carbapenem-resistant *K. pneumoniae* and *E. coli* during this outbreak and 10 demised. The infections however continued until March 2020 (Magobo et al., 2023).

Clinical samples from the hospital’s neonatal unit that had been sent for diagnosis at the National Health Laboratory Services (NHLS), Tshwane Academic Division/Department of Medical Microbiology, University of Pretoria, between September 2019 and March 2020, were therefore collected and analyzed. A series of phenotypic and molecular analyses of the samples were undertaken to delineate the molecular epidemiology and resistance mechanisms of the strains involved in the outbreak. Using whole-genome sequencing and phylogenomics, the evolutionary relationship between the isolates and other regional and global strains as well as the genetic context of their resistance determinants is characterized herein.

## Methods

### Study setting and samples

An uptick in carbapenemase-mediated infection outbreak was observed at the Tembisa hospital in September 2019. We thus followed up on this to trace and reign in the infection from further spread. Forty-five neonatal demographic data, representing 45 patients, and 48 clinical samples from the Tembisa hospital were sent to the National Health Laboratory Services (NHLS), Tshwane Academic Division/Department of Medical Microbiology, University of Pretoria, between September 2019 and March 2020. The samples were collected with sterile swabs and/or sample collection tubes and stored at -80°C freezer. The clinical and demographic data were curated into Microsoft Excel for downstream statistical analysis. The host age, disease, and sample sources (e.g., urine, blood, rectal swab, endotracheal aspirate) were included in the clinical data. Descriptive statistics were used to analyze the demographic data.

### Isolate identification and resistance screening

The samples (48 stored isolates) were retrieved from the -70°C freezer, thawed and plated out on blood agar plates (BAP) (Diagnostic Media Products, South Africa) for further testing. However, only 40 isolates could be revived. Hence, all subsequent phenotypic and PCR tests were done on 40 isolates. The species and antibiotic resistance profiles of the forty isolates were identified using Vitek 2 system (Biomerieux, Johannesburg, South Africa). Furthermore, the carbapenem minimum inhibitory concentrations (MIC) for all isolates were determined with ertapenem, imipenem and meropenem Epsilon tests (E-tests) (BioMèrieux, France). Briefly, a 0.5 McFarland inoculum of each isolate was lawned onto a Mueller-Hinton agar plate (Diagnostic Media Products, South Africa) and the E-test was placed at the center of the plate and incubated in ambient air at 35-37°C for 18-24 hours. The MIC was read at the point where the inhibition ellipse intersects with the test strip using the 2023 CLSI M-100 breakpoints [imipenem, meropenem, and doripenem resistance: ≥ 4 µg/mL; ertapenem resistance: ≥ 2 µg/mL] (CLSI & Clinical and Laboratory Standards Institute (CLSI), 2023).

The colistin MIC for the isolates were determined with broth microdilution (BMD). Colistin BMD was performed according to the Clinical and Laboratory Standards Institute (CLSI) document M07-A10 in an untreated 96-well microtiter polystyrene plate (Clinical and Laboratory Standards Institute (CLSI), 2012). Following subculture of the isolates on BAP’s, a 0.5 McFarland was inoculated into microtiter wells with colistin concentration ranging from 0.125 µg/mL to 64 µg/mL. The microtiter plates were incubated in ambient air at 35-37°C for 18-24 hours. The MIC was read at the first well with no macroscopically visible bacterial growth and interpreted using the 2023 CLSI M-100 breakpoints [Colistin resistance: ≥ 4 µg/mL] (CLSI & Clinical and Laboratory Standards Institute (CLSI), 2023).

The modified carbapenem inactivation method (mCIM) was used to phenotypically screen the isolates for carbapenemase production (Gill et al., 2020). The mCIM is a recently approved phenotypic test for identifying carbapenemase-producing Enterobacteriaceae (CPEs). Briefly, a 10 µg meropenem (MEM) disc was placed aseptically into a suspension of the test CPE isolate and incubated for 4 h ± 15 min at 35°C ± 2°C in ambient air. A suspension of 0.5 McFarland *Escherichia coli* ATCC 25922, a carbapenem-susceptible strain, was plated on a MHA plate. The MEM disk was then removed from the bacterial suspension and placed on the inoculated Mueller-Hinton plate. The plate was incubated for 18 to 24 h at 35°C ± 2°C.

### DNA extraction and PCR

A second subculture on BAP was done to obtain fresh cultures for DNA extraction and PCR to determine the presence of carbapenemase and colistin mobile resistance (*mcr*) genes. The isolates were further screened molecularly using multiplex PCR for *bla*_IMP_, *bla*_KPC_, *bla*_VIM,_ *bla*_OXA_, and *bla*_NDM_ carbapenemases as well as for *Mcr -1, Mcr -2, Mcr -3, Mcr-4*, and *Mcr-5* colistin resistance genes. The PCR screening included one multiplex PCR for *bla_VIM_*, *bla_OXA_* and *bla_NDM_* primers and two singleplex PCRs for both *bla*_KPC_ and *bla*_IMP_.The primers and the conditions used are shown in **Supplementary Table S1**. In-house positive controls for carbapenemases were used while *mcr*-positive controls were provided by the Technical University of Denmark.

*Genomic and bioinformatic analyses*. Owing to funding restrictions, we could only sequence 34 isolates. Therefore, we used the phenotypic resistance profile data of the isolates to select the subset of 34 out of 40 isolates for genomic DNA extraction and sequencing; isolates with resistance to more than three antibiotics were prioritized for the whole-genome sequencing. gDNA were extracted from the isolates using the MagnaPure 96 instrument (Roche, South Africa) from 24-hour BAP cultures. The gDNA were sequenced on an Illumina Miseq at the genomic core sequencing facility of the National Institute of Communicable Diseases (NICD) (Johannesburg, South Africa). The generated fastQ and fastA files were submitted to GenBank under bioproject number PRJNA850834.

The genomes were annotated with NCBIs Prokaryotic Genome Annotation Pipeline (PGAP). The generated.gff files were used to delineate the genetic environment and associated mobile genetic environment of the resistance genes. The species of each of the isolates were confirmed using NCBIs Average Nucleotide Identity (ANI). ResFinder (https://cge.food.dtu.dk/services/ResFinder/) was used to determine the resistance determinants in the genomes. The clonality of the isolates and their multi-locus sequence typing (MLST) numbers were identified using MLST 2.0 (https://cge.food.dtu.dk/services/MLST/) (Larsen et al., 2012). The plasmid replicon genes or incompatibility groups and associated contigs were determined using PlasmidFinder (https://cge.food.dtu.dk/services/PlasmidFinder/). Mobile Element Finder was used to identify the mobile genetic elements (https://cge.food.dtu.dk/services/MobileElementFinder/) in the genomes, while the virulence genes were identified using VirulenceFinder (https://cge.food.dtu.dk/services/VirulenceFinder/).

### Phylogenomics

The genomes of *K. pneumoniae, Enterobacter cloacae, Citrobacter portucalensis*, and *E. coli* isolates that are carbapenem- and/or colistin-resistant were curated from NCBI and PATRIC for purposes of determining their evolutionary relationships with this study’s isolates. These curated genomes were categorized geographically into South Africa, Africa, and global for the phylogenetic analyses. This study’s genomes and those from each of the three categories described above were aligned using Clustalw, which selected and aligned genes and sequences that were common to all aligned isolates. A minimum of 1000 genes from each isolates’ genome were thus aligned for the core genome phylogenetic tree construction. The phylogenies were subsequently inferred using RAxML (using a bootstrap reassessment of 1,000 x) and annotated with Figtree.

The ARGs from this study’s genomes and those of the genomes curated from NCBI and PATRIC were obtained from NCBI or ResFinder (https://cge.food.dtu.dk/services/ResFinder/). These ARGs were tabulated and arranged according to the host isolates’ location on the phylogenetic tree to provide a comparative phylogenomic view of the ARGs per clone or evolutionary distance.

### Ethical approval

This research was approved by the Ethical Review Board of the School of Medicine, University of Pretoria, South Africa.

## Results

### Outbreak settings and demographics

An increase in carbapenem-resistant infections were observed between September 2019 and March 2020 within the neonatal unit 4 at Tembisa hospital, South Africa. This outbreak involved 45 neonates who were born between August 2019 and March 2020 (**Supplementary Table S2**). The affected infants were aged between 1 and 70 days (**Supplementary Table S2**). Blood cultures (n = 26 specimen), and rectal swabs (n = 23 specimen) formed the most common clinical specimens collected from the infants. Single specimens were taken from tracheal aspirate (n = 1), urine (n = 1) and pus swab (n =1). Some of the specimens were taken from the same patient/infant (duplicates), hence the higher number of specimens than that of infants.

### Initial identification and antibiotic sensitivity testing

The isolates from the specimen were initially identified by Vitek 2 as *K. pneumoniae*, with seven specimens having a mixture of both *K. pneumoniae* and *Citrobacter* spp. (n = 2), *Enterobacter* spp. (n = 2), and/or *E. coli* (n = 3). Vitek II susceptibility testing found two isolates to be resistant to colistin with MICs ≥ 64 mg/uL and 19 isolates to be resistant to at least one carbapenem with an MIC of ≥ 2 mg/uL (**Supplementary Table S2**).

*Carbapenemase production, MICs, and PCRs.* The results of mCIM, carbapenems MICs (E-test except for ertapenem for which Vitek 2 was used), colistin BMD, and PCRs are summarized in **Table 1**. All except four of the isolates were resistant to at least one carbapenem (imipenem and/or ertapenem: MICs ranged from 4 to ≥ 32 g/L) from the E-test; yet the four non-resistant isolates harbored a *bla*_OXA-48_-like carbapenemase. There were 31 imipenem- or ertapenem-resistant isolates, and 27 meropenem-resistant isolates while 27 isolates were resistant to all three carbapenems. All except seven isolates were colistin-resistant (MICs: 32 to > 64 g/L); these seven resistant isolates had no *mcr-1* to *-5* genes (**Table 1**).

**Table 1:** Summary of phenotypic, biochemical, and molecular (PCR) results for the carbapenem-resistant strains.

No.	Sample name	Species	MLST^*^	Study number	Specimen	mCIM	Carbapenemase (KPC, OXA, VIM, NDM, IMP)	Carbapenem MICs	Col BMD mg/L	Mcr 1-5
1	JSK_2019-1	*Klebsiella pneumoniae*	ST5785	T1	Rectal swab	+^^†^^	OXA+VIM	I^‡^=0.5, M^§^=0.5, E^††^=>32	0.25	-^^††^^
2	JSK_2019-2	*Klebsiella pneumoniae*	ST25	T2	Rectal swab	+	OXA	I=1, M=2, E=4	0.25	–
3				T3	Rectal swab	+				NG^^‡‡^^
4	JSK_2019-3			T4	Rectal swab	+	OXA	I=1, M=0.5, E=0.5	0.25	
5	JSK_2019-4	*Escherichia coli*	ST5907(1) ST906 (2)	T5	Rectal swab	+	OXA	I=2, M=0.5, E=0.5	0.125	–
6	JSK_2019-5	*Klebsiella pneumoniae*	ST307	T6	Rectal swab	+	OXA	I=>32, M=>32, E=>32	32	–
7	JSK_2019-6	*Klebsiella pneumoniae*	ST297	T7	Rectal swab	+	OXA+NDM	I=>32, M=>32, E=>32	0.125	–
8	JSK_2019-7	*Klebsiella pneumoniae*	ST152	T8	Pus swab	+	OXA+NDM	I=32, M=4, E=32	0.5	–
9	JSK_2019-8	*Klebsiella pneumoniae*	ST307	T9	Rectal swab	+	OXA	I=>32, M=>32, E=>32	32	–
10	JSK_2019-9	*Klebsiella pneumoniae*	ST307	T10	Blood culture	+	OXA	I=>32, M=>32, E=>32	>64	–
11	JSK_2019-10	*Escherichia coli*	ST58	T11	Rectal swab	+	OXA	I=>32, M=1, E=16	0.25	–
12	JSK_2019-11	*Klebsiella pneumoniae*	ST307	T12	Rectal swab	+	OXA	I=32, M=>32, E=>32	>64	–
13	JSK_2019-12	*Enterobacter cloacae*	ST1266	T13	Rectal swab	+	OXA	I=>32, M=>32, E=>32	>64	–
14	JSK_2019-13	*Klebsiella pneumoniae*	ST152	T14	Blood culture	+	NDM	I=>32, M=>32, E=>32	2	–
15	JSK_2019-14	*Klebsiella pneumoniae*	ST307	T15	Urine	+	OXA	I=>32, M=>32, E=>32	0.25	–
16	JSK_2019-15	*Klebsiella pneumoniae*	ST152	T16	Rectal swab	+	NDM	I=>32, M=>32, E=>32	0.125	–
17	JSK_2019-16	*Klebsiella pneumoniae*	ST152	T17	Blood culture	+	NDM	I=>32, M=>32, E=>32	0.125	–
18	JSK_2019-17	*Klebsiella pneumoniae*	ST152	T18	Rectal swab	+	OXA+NDM	I=>32, M=>32, E=>32	0.125	–
19	JSK_2019-18	*Klebsiella pneumoniae*	ST307	T19	Rectal swab	+	OXA	I=>32, M=>32, E=>32	0.125	–
20	JSK_2019-19	*Enterobacter cloacae*	ST1266	T20	Rectal swab	+	OXA	I=>32, M=>32, E=>32	0.125	–
21	JSK_2019-20	*Klebsiella pneumoniae*	ST307	T21	Rectal swab	+	No gene	I=>32, M=1, E=>32	>64	–
22	JSK_2019-21	*Citrobacter portucalensis*	ST248	T22	Rectal swab	+	OXA	I=>32, M=>32, E=>32	0.25	–
23	JSK_2019-22	*Klebsiella pneumoniae*	ST152	T23	Blood culture	+	NDM	I=2, M=4, E=>32	0.25	–
24	JSK_2019-23	*Klebsiella pneumoniae*		T24	Blood culture	+	NDM	I=>32, M=>32, E=>32	0.5	–
25				T25	Rectal swab	+				NG
26				T26	Rectal swab	+				NG
27	JSK_2019-24	*Klebsiella pneumoniae*	ST152	T27	Rectal swab	+	NDM	I=>32, M=>32, E=>32	0.125	–
28	JSK_2019-25	*Klebsiella pneumoniae*	ST152	T28	Blood culture	+	NDM	I=>32, M=>32, E=>32	0.125	–
29	JSK_2019-26	*Klebsiella pneumoniae*	ST152	T29	Blood culture	+	NDM	I=>32, M=>32, E=>32	0.125	–
30	JSK_2019-27	*Klebsiella pneumoniae*	ST152	T30	ETA	+	NDM	I=>32, M=>32, E=>32	1	–
31	JSK_2019-28	*Klebsiella pneumoniae*	ST152	T31	Blood culture	+	NDM	I=>32, M=>32, E=>32	0.25	–
32	JSK_2019-29	*Klebsiella pneumoniae*	ST152	T32	Blood culture	+	NDM	I=>32, M=>32, E=>32	0.25	–
33	JSK_2019-30	*Klebsiella pneumoniae*	ST152	T33	Blood culture	+	NDM	I=>32, M=4, E=>32	0.125	–
34	JSK_2019-31	*Klebsiella pneumoniae*		T34	Blood culture	+	NDM	I=>32, M=>32, E=>32	0.125	–
35				T35	Rectal swab	+				NG
36				T36	Blood culture	+			0.25	NG
37	JSK_2019-32	*Klebsiella pneumoniae*	ST307	T37	Blood culture	+	OXA	I=>32, M=>32, E=>32	0.125	–
38	JSK_2019-33	*Klebsiella pneumoniae*	ST307	T38	Rectal swab	+	OXA	I=>32, M=>32, E=>32	0.125	–
39	JSK_2019-34	*Klebsiella pneumoniae*	ST17	T39	Blood culture	+	OXA	I=>32, M=>32, E=>32	0.125	–
40	JSK_2019-35	*Klebsiella pneumoniae*	ST17	T40	Blood culture	+	OXA	I=1, M=0.25, E=0.5	0.25	–
41	JSK_2019-36	*Klebsiella pneumoniae*		T41	Rectal swab	+	NDM	I=32, M=16, E=O/S (no Vitek MIC)	0.125	–
42	JSK_2019-37	*Klebsiella pneumoniae*		T42	Rectal swab	+	NDM	I=>32, M=>32, E=O/S (no Vitek MIC)	0.125	–
43				T43	Rectal swab	+				NG
44				T44	Rectal swab	+				NG
45	JSK_2019-38	*Klebsiella pneumoniae*		T45	Rectal swab	+	OXA	I=1, M=0.25, E=0.5	0.25	–
46	JSK_2019-39	*Enterobacter cloacae*	ST1266	T46	Rectal swab	+	OXA	I=4, M=16, E=O/S (no Vitek MIC)	>64	–
47				T47	Rectal swab	+				NG
48	JSK_2019-40	*Escherichia coli*	ST457(1) ST829(2)	T48	Rectal swab	+	OXA	I=16, M=2, E=O/S (no Vitek MIC)	0.25	–

* Multi-locus sequence typing.

† Positive results.

‡ Imipenem MIC.

§ Meropenem MIC.

** Ertapenem MIC.

†† Negative PCR results.

‡‡ No growth.

All the isolates were positive for the mCIM and PCR carbapenemase tests, except T21 (JSK_2019-20), which was mCIM-positive, but PCR-negative. There were 15 NDM-positive isolates, 18 OXA-48–like–positive isolates, three NDM + OXA-48–like–positive isolates, and one VIM + OXA-48–like–positive isolates. None of the isolates was *mcr-1* to -5–positive. Compared with OXA-48–like–positive isolates, all NDM-positive isolates were resistant to all three carbapenems except T33 (meropenem MIC = 4 µg/mL), T23 (imipenem MIC = 24 µg/mL; meropenem MIC = 4 µg/mL), and T8 (OXA-48–like and NDM-positive; meropenem MIC = 4 µg/mL); see **Table 1**.

### Genomic analyses

#### Identification and resistance profiles

The species of the isolates were confirmed by their average nucleotide identity (ANI) on NCBI. Of the 34 sequenced isolates, 27 were *Klebsiella pneumoniae*, three were *E. coli* (T-5, T-11, and T-48), three were *E. cloacae* (T-13, T-20, and T-46), and one was *Citrobacter portucalensis* (T-22). The inferred resistance profiles of the isolates, from their resistance genes, showed that 33/34 isolates were multidrug resistant. Except *E. cloacae* (T13, T20, and T46) and T48 (*E. coli*), all the other isolates were resistant to at least four antibiotic classes: aminocyclitol, aminoglycosides, amphenicol, β-lactam, folate pathway antagonist, fosfomycin, macrolide, peroxide, quaternary ammonium compound, quinolone, rifamycin, and tetracycline (**Supplementary Table S3**). Only one isolate had a tetracycline resistance gene.

#### Resistance determinants

The distribution of the antibiotic resistance genes per isolate is shown in **Supplementary Table S3**, with the most frequently identified being *oqxAB* (fluoroquinolones resistance: n = 28 isolates), *fosA* (fosfomycin resistance: n = 27 isolates), *sul-1* & *sul-2* (sulphonamide resistance: n = 23 isolates), *qacE* (biocide resistance: n = 22 isolates), *mphA* (macrolide resistance: n = 20 isolates), *aac(6’)-Ib-cr* (fluoroquinolone/aminoglycoside resistance: n = 20 isolates), *aph(3”)-Ib* (aminoglycoside resistance: n = 17 isolates), *aph(6)-Id* (aminoglycoside resistance: n = 17 isolates), *aadA16* (aminoglycoside resistance: n = 16 isolates)*, dfrA27* (trimethoprim resistance: n = 16 isolates), *arr-3* (rifamycin resistance: n = 16 isolates), *bla*_CTX-M-15_ (β-lactam resistance: n = 16 isolates), *bla*_TEM-1B_ (β-lactam resistance: n = 16 isolates), *bla*_OXA-48_ (β-lactam resistance: n = 15 isolates), *bla*_SHV-187_ (β-lactam resistance: n = 14 isolates), and *rmtC* (aminoglycoside resistance n = 12 isolates). *bla*_NDM-1_ (β-lactam resistance) was present in 13 isolates (**Supplementary Table S3**).

Common mutation-based resistance mechanisms were found in *gyrA* (n = 25 isolates), *ompK36* (n = 26 isolates), and *ompK37* (n = 26 isolates). Nineteen *K. pneumoniae* isolates viz., T8, T9, T10, T12, T14, T16, T17, T18, T19, T23, T27, T28, T29, T30, T31, T32, T33, T37, and T39, had between 15 and 22 resistance genes (**Supplementary Table S3**).

Discrepancies between the PCR data and the whole-genome sequencing (WGS) data were observed. PCR did not identify *bla*_OXA-181_ in strain T21 (JSK-2019-20), albeit WGS identified this gene and mCIM showed the presence of a carbapenemase. Moreover, the WGS data did not confirm the presence of VIM + OXA-48, NDM + OXA-48, and the presence of OXA and NDM in some of the isolates. Indeed, some of the isolates could not be revived for WGS after the PCR screening step. Evidently, some might have lost their plasmids during the subsequent culturing steps to obtain 24-hour genomic DNA for the WGS. However, the presence of both NDM and OXA-48/181 in the isolates were mostly confirmed by both PCR and WGS (**Table 1**; **Supplementary Table S3**).

*Virulence factors.* Among the 34 sequenced isolates, 24 virulence genes were identified: *air* (n = 1 isolate), *ccl* (n = 12 isolates), *cia* (n = 2 isolates), *chuA* (n = 2 isolates), *cvaC* (n = 1 isolate), *eilA* (n = 2 isolates), *etsC* (n = 1 isolate), *fyuA* (n = 18 isolates), *gad* (n = 2 isolates), *hylF* (n = 1 isolate), *KpsMII_K5* (n = 1 isolate), *KpsE* (n = 1 isolate), *ipfA* (n = 2 isolates), *iroN* (n = 1 isolates), *irp2* (n = 18 isolates), *iss* (n = 2 isolates), *iucC* (n = 1 isolate), *iutA* (n = 28 isolates), *mchF* (n = 1 isolate), *ompT* (n = 2 isolates), *sitA* (n = 1 isolate), *terC* (n = 12 isolates), *traT* (n = 5 isolates), and *yfcV* (n = 1 isolate). *E. coli* isolates T11 and T48 had the highest number of virulence genes (n = 17 and 13, respectively), while the other isolates harbored between one and four genes.

#### Plasmid replicon/incompatibility types

An average of four plasmid incompatibility groups were found in each isolate, with higher numbers being detected in isolates T1 (n = 7), T2 (n = 9), T11 (n = 5), T18 (n = 8), T20 (n = 5), T22 (n = 6), T39 (n = 6), and T40 (n = 6). The remaining isolates had between one and four incompatibility groups, with four plasmid types per isolate being very dominant. There were 25 plasmid types, of which IncFIA(HI1) (n = 5 plasmid types), Col(pHAD28) (n = 8 plasmid types), ColKP3 (n = 10 plasmid types), ColRNAI (n = 14 plasmid types), IncFIB(K) (n = 15 plasmid types), IncFIB(pNDM-Mar) (n = 7 plasmid types), IncFII(Yp) (n = 16 plasmid types), IncL (n = 16 plasmid types) and IncX3 (n = 10 plasmid types) were most common.

Of the 55 mobile genetic elements (MGEs) identified in the isolates, *IS*Vsa3 (n = 5 isolates), *IS*Ec15 (n = 7 isolates), *IS*Ec36 (n = 10 isolates), *IS*Kpn19 (n = 11 isolates), *IS*Kpn21 (n = 11 isolates), *IS*Kpn26 (n = 11 isolates), *IS*Kpn28 (n = 11 isolates), *IS*Kpn34 (n = 11 isolates), *IS*Kpn43 (n = 11 isolates), *IS*Sen4 (n = 11 isolates), *IS*26 (n = 12 isolates), *IS*Ec33 (n = 13 isolates), *IS*Kox1 (n = 13 isolates), *IS*5075 (n = 19 isolates), *IS*6100 (n = 22 isolates), *IS*Kox3 (n = 24 isolates), and *IS*Kpn1 (n = 20 isolates) were prominent. The number of MGEs per isolate ranged from three to 16, with an average of nine MGEs per isolate.

**Figure 1:**
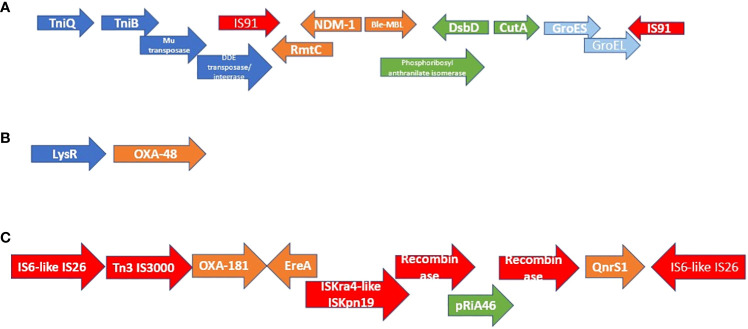
Genetic environment and synteny of mobile genetic elements associated with NDM-1, OXA-84, and OXA-181. The genetic environment of NDM-1 is shown in figure **(A)** while those of OXA-48 and OXA-181 are shown in figures **(B, C)**. All the NDM-positive isolates had the same synteny and genetic environment within the immediate flanks of NDM-1. In six cases, the order was reversed (in orientation) but the composition remained the same. OXA-48:LysR was also found in all OXA-48–positive isolates in the same or reverse orientation. All isolates that were OXA-181–positive also had the same, reversed orientation, or truncated version of the genetic environment shown in **(C)**.

The MGEs associated with *bla*_NDM-1_ and *bla*_OXA-48/-181_ are shown in **Figure 1**. Irrespective of the strain (MLST) or species, the same genetic environment in the same or reversed orientation, or a truncated version of it (in the case of OXA-181), was found within the immediate flanks of *bla*_NDM-1_, *bla*_OXA-48_, and *bla*_OXA-181_ (**Figure 1**).

The clonality of the isolates was determined using their MLST (see **Table 1**; **Supplementary Table S3**). All the three *E. coli* strains were not related and three of the *K. pneumoniae* strains (i.e., ST5785, ST25, and ST297) were also singletons with no relation with the other *K. pneumoniae* strains that occurred in multiples: ST17 (n = 2); ST1266 (n = 3); ST307 (n = 9); ST152 (n = 13). Whereas the single *K. pneumoniae, E. coli*, and *C. portucalensis* clones as well as the three *E. cloacae* clones were isolated in 2020 from rectal swabs, *K. pneumoniae* ST307 and ST152 were identified between 2019 and 2020 from different specimen types: pus, ETA, rectal swab, blood culture, and urine. *K. pneumoniae* ST17 was only isolated in 2019 from blood cultures only.

A uniformity in resistance profiles, ARGs, virulence genes, plasmid types, and MGEs was not observed among isolates of the same clone although some genes and genetic elements were uniformly present in members of the same clone (**Supplementary Table S3**).

#### Phylogenomics

**Figure 2:**
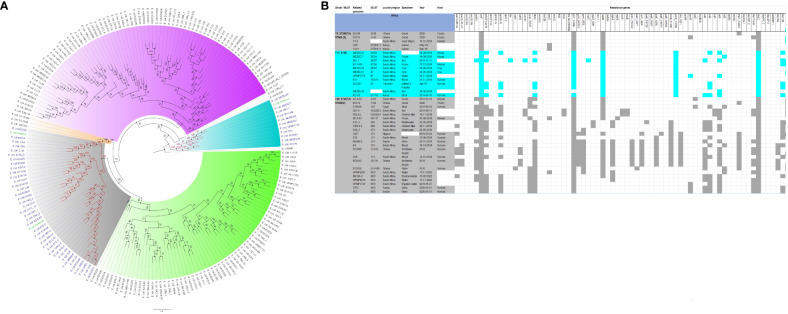
Phylogenomic and comparative resistance genes’ profile analyses of *E. coli* strains involved in the outbreak and other strains of the same clone from Africa. The comparative phylogenetic analyses of the *E. coli* strains involved in this outbreak and other strains of the same clone from Africa are shown in **(A)**. The names of the strains from this study are coloured in green. The names of closely related strains to this study’s strains are shown in blue. Branches holding this study’s strain with very high bootstrap values (>50%) are shown in red to depict isolates with very close evolutionary distance. The resistance genes found in each strain in **(A)** is shown in **(B)** with the demographics of the host.

**Figure 3:**
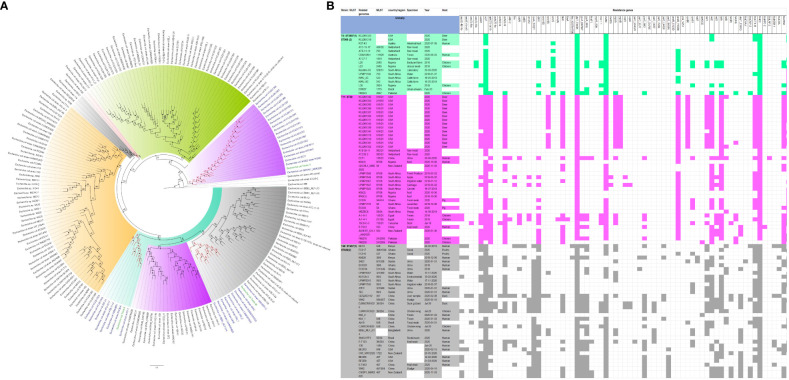
Phylogenomic and comparative resistance genes’ profile analyses of *E. coli* strains involved in the outbreak and other strains of the same clone from the whole world (global). The comparative phylogenetic analyses of the *E. coli* strains involved in this outbreak and other strains of the same clone from the whole world are shown in **(A)**. The names of the strains from this study are coloured green. The names of closely related strains to this study’s strains are shown in blue. Branches holding this study’s strain with very high bootstrap values (>50%) are shown in red to depict isolates with very close evolutionary distance. The resistance genes found in each strain in **(A)** is shown in **(B)** with the demographics of the host.

**Figure 4:**
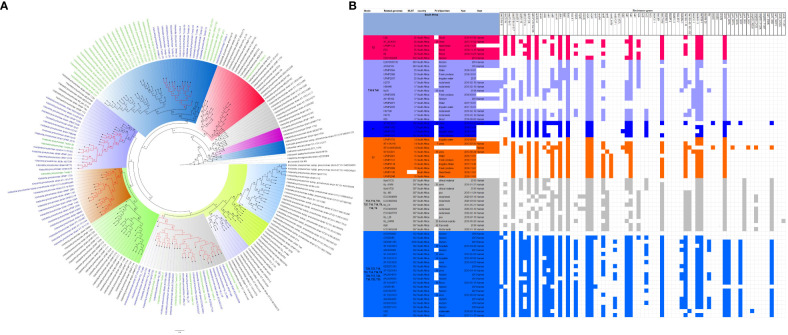
Phylogenomic and comparative resistance genes’ profile analyses of *K. pneumoniae* strains involved in the outbreak and other strains of the same clone from South Africa. The comparative phylogenetic analyses of the *K. pneumoniae* strains involved in this outbreak and other strains of the same clone from South Africa are shown in **(A)**. The names of the strains from this study are coloured green. The names of closely related strains to this study’s strains are shown in blue. Branches holding this study’s strain with very high bootstrap values (>50%) are shown in red to depict isolates with very close evolutionary distance. The resistance genes found in each strain in **(A)** is shown in **(B)** together with the demographics of the host.

**Figure 5:**
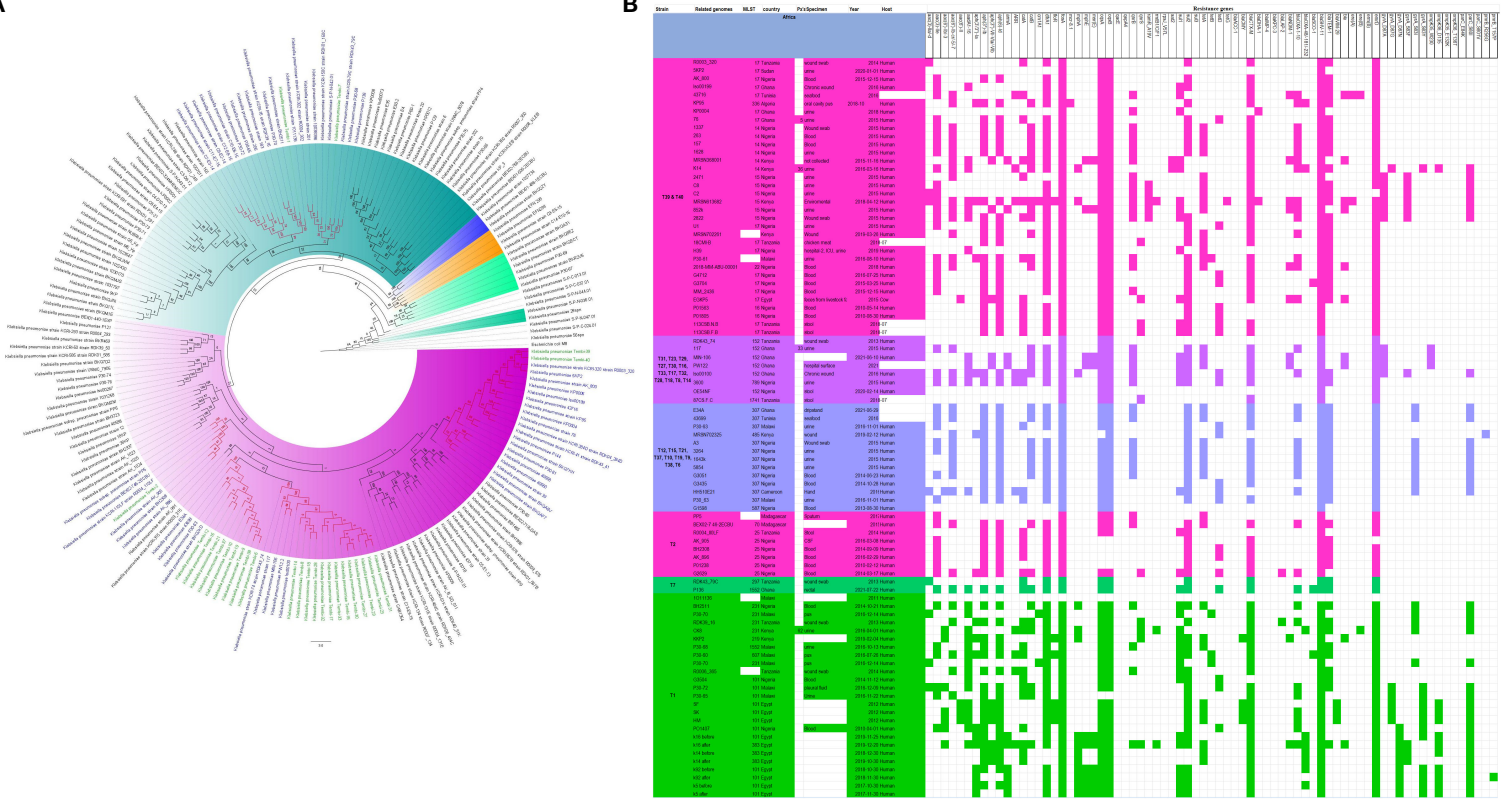
Phylogenomic and comparative resistance genes’ profile analyses of *K. pneumoniae* strains involved in the outbreak and other strains of the same clone from Africa. The comparative phylogenetic analyses of the *K. pneumoniae* strains involved in this outbreak and other strains of the same clone from Africa are shown in **(A)**. The names of the strains from this study are coloured green. The names of closely related strains to this study’s strains are shown in blue. Branches holding this study’s strain with very high bootstrap values (>50%) are shown in red to depict isolates with very close evolutionary distance. The resistance genes found in each strain in **(A)** is shown in **(B)** together with the demographics of the host.

**Figure 6:**
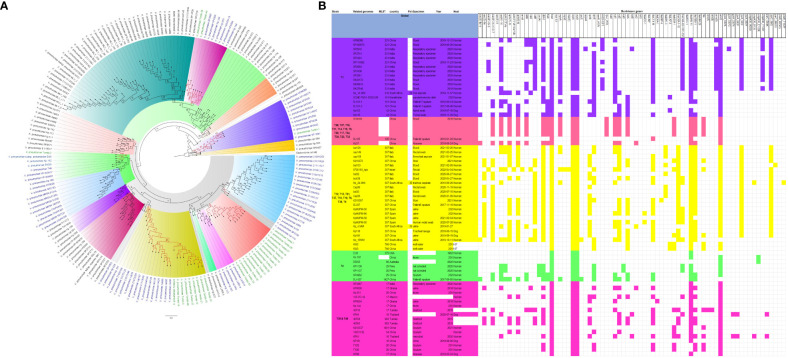
Phylogenomic and comparative resistance genes’ profile analyses of *K. pneumoniae* strains involved in the outbreak and other strains of the same clone from the world. The comparative phylogenetic analyses of the *K. pneumoniae* strains involved in this outbreak and other strains of the same clone from the world are shown in **(A)**. The names of the strains from this study are coloured green. The names of closely related strains to this study’s strains are shown in blue. Branches holding this study’s strain with very high bootstrap values (>50%) are shown in red to depict isolates with very close evolutionary distance. The resistance genes found in each strain in **(A)** is shown in **(B)** together with the demographics of the host.

**Figure 7:**
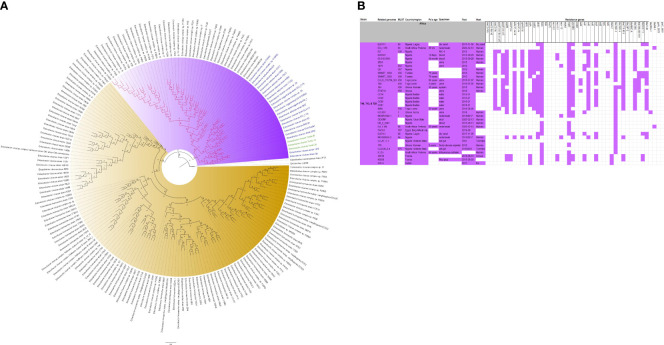
Phylogenomic and comparative resistance genes’ profile analyses of *E. cloacae* strains involved in the outbreak and other strains of the same clone from Africa. The comparative phylogenetic analyses of the *E. cloacae* strains involved in this outbreak and other strains of the same clone from Africa are shown in **(A)**. The names of the strains from this study are coloured green. The names of closely related strains to this study’s strains are shown in blue. Branches holding this study’s strain with very high bootstrap values (>50%) are shown in red to depict isolates with very close evolutionary distance. The resistance genes found in each strain in **(A)** is shown in **(B)** together with the demographics of the host.

**Figure 8:**
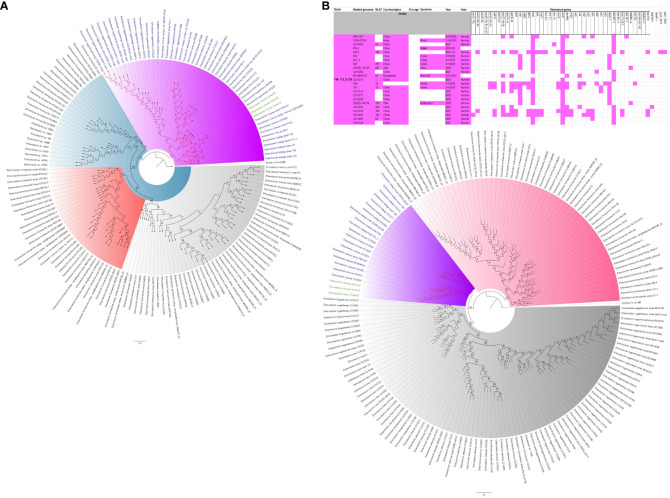
Phylogenomic and comparative resistance genes’ profile analyses of *E. cloacae* strains involved in the outbreak and other strains of the same clone from the world. The comparative phylogenetic analyses of the *E. cloacae* strains involved in this outbreak and other strains of the same clone from the world are shown in **(A)**. The names of the strains from this study are coloured green. The names of closely related strains to this study’s strains are shown in blue. Branches holding this study’s strain with very high bootstrap values (>50%) are shown in red to depict isolates with very close evolutionary distance. The resistance genes found in each strain in **(A)** is shown in **(B)** together with the demographics of the host.

The evolutionary relationships and antibiotic resistance patterns between this study’s isolates and other closely related isolates are depicted in **Figures 2**–**8**; **Supplementary Figures S1**–**S7**, and **Supplementary Table S4**. With some exceptions, isolates belonging to the same MLST (clones) were found to be closely related (found on the same branch with bootstrap values of more than 50) to each other than those from different MLST clones on the trees. Moreover, the resistance profiles of the clones/isolates found on the same branch (with very close evolutionary distance) were very similar, albeit some differences were also observed. For instance, irrespective of country, source, and date of isolation, *E. coli* ST131 clones had very similar ARG profiles (**Figure 2**), suggesting a possible international transmission of that clone with their associated ARGs. Similar trends were also observed in closely related *K. pneumoniae* clones and isolates on the same cluster or branch. Notably, *E. cloacae* isolates that were of very close evolutionary distance in **Figure 7** had very similar resistance profiles, which differed from those that were distant and found on different branches and clusters. These uniform patterns were observed in strains that were isolated from different parts of the world and from different clinical and environmental specimen types.

## Discussion

In this study, we describe an outbreak of fatal carbapenem-resistant *K. pneumoniae, E. coli*, and *E. cloacae* infections in a tertiary hospital in Gauteng, South Africa, which contributed to the mortality of at least 10 neonates. It is worrying to note that the strains were mostly multidrug-resistant, with all except four of the strains being resistant to at least four antibiotic classes (**Figures 2**-**8**; **Supplementary Figure S1**-**S7**-**S9**; **Supplementary Tables 1**, **Supplementary Figure S1**-**S4**). More concerning is the presence of carbapenemases in all the strains, which confer resistance to almost all β-lactams. Although no *mcr* genes were identified, seven strains were resistant to colistin, a last-resort antibiotic for carbapenem-resistant Gram-negative bacterial infections (Ramaloko and Osei Sekyere, 2022). Other non-*mcr* colistin resistance mechanisms in *Enterobacteriaceae* have been identified (including *pmrAB*, *phoPQ*, *crrAB* and *mgrB* mutations) and any of these mechanisms could be responsible for the colistin resistance identified in these isolates.

Outbreaks involving *K. pneumoniae* and *E. coli* in hospital settings are common; including fatal outbreaks in neonatal units as reported in this study (Osei Sekyere et al., 2021). Indeed, this is not the first outbreak to occur in South Africa as well. However, this study presents one of the most comprehensive molecular and genomic analyses of infection outbreaks involving these species and *E. cloacae* (Osei Sekyere and Reta, 2020b).

Although all the isolates were mCIM-positive and all-but-one was PCR-positive for carbapenemases, they were not all phenotypically resistant to carbapenems; at least four isolates were carbapenem-susceptible (**Table 1**). This observation is not singular as some isolates with carbapenemases have been reported to be phenotypically susceptible to carbapenems. For such observations, it was concluded that the carbapenemases were not expressed, although they were present (Nordmann and Poirel, 2019; Kopotsa et al., 2020). For the PCR-negative but mCIM-positive isolate, there could be the presence of either a novel carbapenemase or a known carbapenemase that was not targeted by the PCR. The latter possibility was invalidated by the presence of *bla*_OXA-181_, which was identified by the whole-genome data but was not identified by the PCR. Thus, the mCIM agreed with the whole-genome sequencing data.

However, it is worth noting that carbapenem resistance is not only mediated by carbapenemases as other determinants such as hyperexpression of AmpCs, ESBLS, and efflux pumps and hypo-expression of porins also determine carbapenem resistance (Queenan and Bush, 2007; Osei Sekyere et al., 2016; Mmatli et al., 2020). The presence of a resistance gene with a non-commensurate phenotypic expression of the resistance gene is not limited to carbapenems alone. This is one of the reasons why molecular diagnostic tests for antibiotic resistance genes cannot be used solely as representative or confirmatory of phenotypically resistant pathogens. In this study, we also observed that the colistin-resistant isolates did not have *mcr* genes (Mmatli et al., 2020; Ramaloko and Osei Sekyere, 2022).

As recently observed in a molecular study to screen for carbapenemases and *mcr* genes, *mcr* genes are relatively less prevalent than carbapenemases (Mmatli et al., 2022a). This phenomenon was also observed in this study as no *mcr* gene was identified, albeit seven isolates were colistin-resistant. This is a positive finding as colistin is currently the last-resort antibiotic for carbapenem-resistant infections (Mmatli et al., 2022b; Ramaloko and Osei Sekyere, 2022). Moreover, the rarity of *mcr* genes will help reduce the spread of colistin resistance as *mcr* genes are mainly plasmid-borne and very mobile.

Similar findings with higher prevalence of *bla*_OXA-181/48_ and *bla*_NDM_ carbapenemase genes in Gram-negative bacteria has been reported in South Africa, confirming this observation, and showing that these two carbapenemases are common in South African hospitals (Pedersen et al., 2018; Lowe et al., 2019; Choi et al., 2020; Kopotsa et al., 2020; Perovic et al., 2020; Lowe et al., 2022). The phylogenomic analyses of the South African isolates confirm these observations and similar patterns (**Supplementary Table S4**). Furthermore, the presence of other clinically important resistance genes such as *bla*_CTX-M-15_, *bla*_SHV_, *bla*_TEM_, *qnrB/S, oqxA/B, dfrA, Sul1/2/3, aac(6’)-lb-cr, aadA*, and *mph(A)* explains the multidrug and pan-drug resistance nature of the isolates. This might explain the failure of the clinicians to treat these infections, leading to the mortality of 10 infants.

As shown in **Figure 1**, the carbapenemases in the isolates were flanked by other resistance genes and mobile genetic elements such as composite transposons: *IS*91 (*bla*_NDM_) and *IS*6-like *IS*26 (*bla*_OXA-181_). Instructively, irrespective of the strain in which any of the three carbapenemase i.e., *bla*_OXA-48_, *bla*_OXA-181_, and *bla*_NDM-1_, was found, their genetic support/environment were all the same. This similarity in synteny and flanks around these resistance genes is further corroborated by the fact that most of these isolates, irrespective of the species or the clone, had the same insertion sequences: *IS*26 (n = 12); *IS*Ec33 (n = 13); *IS*Kpn26 (n = 16); *IS*Sen4 (n = 16); *IS*5075 (n = 19); *IS*Kpn1 (n = 20); *IS*6100 (n = 22); *IS*Kox3 (n = 24) (**Supplementary Table S3**: MGEs). Furthermore, each of the isolate had an average of three plasmid replicons/incompatibilities, with the least being one and the highest being nine. The commonest of these plasmid types among all the isolates were IncL (n = 16), IncFII(Yp) (n = 16), IncFIB(K) (n = 15), ColRNAI (n = 14), IncX_3_ (n = 10), and ColKP3 (n = 10). Notably, IncL, IncF, and IncX_3_ plasmids are associated with important β-lactam resistance genes such as *mcr, bla*_CTX-M-15_, *bla*_OXA-48_, *bla*_OXA-181_, and *bla*_NDM-1_, and are implicated in the dissemination of these resistance genes between different Enterobacteriaceae species. Particularly, IncF plasmids have high-frequency transfer ability, which contributes to high dissemination of resistance genes among bacteria (Kopotsa et al., 2019; Kopotsa et al., 2020; Mmatli et al., 2022b).

Owing to the break-up of the genomes into several contigs, it is difficult to associate these plasmid types with each ARG. However, taken together, these show that the ARGs in these MDR isolates are being disseminated through these MGEs (**Supplementary Table S3**: Plasmid incompatibility). These genetic environments and ARG flanks are also commonly observed in other studies involving the same or different species within Enterobacterales from both South Africa and globally, corroborating the role of MGEs in the transmission of carbapenemases and other ARGs within and across clones and species (Kopotsa et al., 2019; Kopotsa et al., 2020; Osei Sekyere and Reta, 2020a; Osei Sekyere and Reta, 2020b; Ramaloko and Osei Sekyere, 2022).

Although there are differences in the ARGs present in the isolates (**Supplementary Table S3**: ARGs), there are very close similarities and uniformity in the ARGs present in the isolates. This uniformity in ARGs, MGEs, and plasmid incompatibility/replicon types across the isolates suggest that irrespective of the clones and species, there are plasmid-borne MGEs that are shuttling the ARGs between and across the species and clones (Kopotsa et al., 2019; Kopotsa et al., 2020; Osei Sekyere and Reta, 2020b; Ramaloko and Osei Sekyere, 2022). Therefore, there is both clonal and plasmid-mediated transmission of the same ARGs in the hospital, particularly when the outbreak occurred in two major wards: ward 4 and 4a-NICU.

In fact, this observation is also magnified in **Figures 2**-**8**, where the same ARGs were seen among isolates of the same clone and isolates with very close evolutionary distance. In particular, the phylogenomic analyses of the isolates show that the same ARGs, possibly hosted on MGEs, and clones are being disseminated across South Africa, Africa, and globally. It is observed from **Figures 2**-**8** that the same clones among the three species cluster together closely in the phylogenetic tree alongside other clones. The presence of other clones clustering alongside the same clones is not surprising given the higher resolution of whole-genome-based typing compared to multi-locus sequence typing (MLST) that only uses seven house-keeping genes. The *E. coli* isolates from this outbreak were of the same clone as *E. coli* clones ST58, ST69, ST87, ST103, ST131, ST457, ST616, and ST648, which were common among other isolates from South Africa, Africa, and globally. The resistance profiles within each of these clones or phylogenetic cluster were mostly uniform with multidrug resistance (**Figures 2**, **3**).

The *K. pneumoniae* clones in this study shared the same clone and phylogenetic cluster as *K. pneumoniae* ST14, ST15, ST17, ST25, ST101, ST152, ST231, and ST307, and the resistance profiles within each of these clonal clusters were very similar with multiple resistance genes (**Figures 4**-**6**). Likewise, the *E. cloacae* isolates clustered with *E. cloacae* ST84 and ST456 with very similar multi-resistance profiles (**Figures 7**, **8**). These observations support the fact that there is clonal and MGE-mediated transmission of these ARGs across the globe, which caused the outbreak in the hospital under study. The study was limited by the relatively smaller number (< 40) of isolates, which was determined by the number of isolates obtained from the collected clinical samples. We were also unable to check the transferability of the ARGs and plasmids from our isolates to *E. coli* owing to budgetary limitations. Future studies can check the transferability of the ARGs identified in these isolates through plasmid transformation experiments.

In summary, there was a carbapenemase-producing Enterobacteriaceae-mediated outbreak in Tembisa hospital that took the lives of 10 neonates. The pathogens (*Klebsiella pneumoniae, Enterobacter cloacae*, and *Escherichia coli*) involved in this outbreak were both clonal and multiclonal. Notably, the strains were of the same clone as other local South African strains and international clones, suggesting that the same clones are spreading these carbapenemases globally. The resistance genes were also found on IncX, IncF, and IncL high-frequency plasmids and bracketed by insertion sequences and transposons that also facilitated a horizontal transmission of the ARGs between strains and species. The combination of both vertical and horizontal transmission of these ARGs explain their rapid spread and high fatality rate (10/45 patients died) among the neonates.

## Data availability statement

The data presented in the study are deposited in the DDBJ/ENA/GenBank repository, accession (bioproject) number PRJNA850834. This Whole Genome Shotgun project has been deposited at DDBJ/ENA/GenBank under the accession JANITZ000000000 to JANIVG000000000. The version described in this paper is version JANITZ010000000 to JANIVG010000000.

## Ethics statement

The studies involving humans were approved by Ethical Review Board of the School of Medicine, University of Pretoria, South Africa. The studies were conducted in accordance with the local legislation and institutional requirements. The human samples used in this study were acquired from a by- product of routine care or industry. Written informed consent for participation was not required from the participants or the participants’ legal guardians/next of kin in accordance with the national legislation and institutional requirements.

## Author contributions

JO: Conceptualization, Data curation, Formal analysis, Funding acquisition, Investigation, Methodology, Project administration, Resources, Software, Supervision, Validation, Visualization, Writing – original draft, Writing – review & editing. MM: Data curation, Investigation, Methodology, Writing – review & editing. AB: Data curation, Investigation, Methodology, Writing – review & editing. RN: Conceptualization, Data curation, Investigation, Methodology, Writing – review & editing. HN: Conceptualization, Data curation, Investigation, Methodology, Project administration, Writing – review & editing. SD: Data curation, Methodology, Writing – review & editing. NEM: Data curation, Writing – review & editing. NMM: Writing – review & editing. MS: Conceptualization, Data curation, Investigation, Methodology, Project administration, Writing – review & editing.

## Supplementary material

Supplementary Figure 1 Comparative phylogenomics of *Escherichia coli* strains from both this study and other strains from South Africa. The names of the strains from this study are colored green. The names of closely related strains to this study’s strains are shown in blue. Branches holding this study’s strain with very high bootstrap values (>50%) are shown in red to depict isolates with very close evolutionary distance.

Supplementary Figure 2 Comparative phylogenomics of *Escherichia coli* strains from both this study and other strains from Africa. The names of the strains from this study are colored green. The names of closely related strains to this study’s strains are shown in blue. Branches holding this study’s strain with very high bootstrap values (>50%) are shown in red to depict isolates with very close evolutionary distance.

Supplementary Figure 3 Comparative phylogenomics of *Escherichia coli* strains from both this study and other strains from the world. The names of the strains from this study are colored green. The names of closely related strains to this study’s strains are shown in blue. Branches holding this study’s strain with very high bootstrap values (>50%) are shown in red to depict isolates with very close evolutionary distance.

Supplementary Figure 4 Comparative phylogenomics of *Klebsiella pneumoniae* strains from both this study and other strains from Africa. The names of the strains from this study are colored green. The names of closely related strains to this study’s strains are shown in blue. Branches holding this study’s strain with very high bootstrap values (>50%) are shown in red to depict isolates with very close evolutionary distance.

Supplementary Figure 5 Comparative phylogenomics of *Klebsiella pneumoniae* strains from both this study and other strains from the world. The names of the strains from this study are colored green. The names of closely related strains to this study’s strains are shown in blue. Branches holding this study’s strain with very high bootstrap values (>50%) are shown in red to depict isolates with very close evolutionary distance. Both S5A and S5B are phylogenetic analyses of global strains.

Supplementary Figure 6 Comparative phylogenomics of *Enterobacter cloacae* strains from both this study and other strains from Africa. The names of the strains from this study are colored green. The names of closely related strains to this study’s strains are shown in blue. Branches holding this study’s strain with very high bootstrap values (>50%) are shown in red to depict isolates with very close evolutionary distance.

Supplementary Figure 7 Comparative phylogenomics of *Citrobacter portucalensis* strains from both this study and other strains from the whole world. The names of the strains from this study are colored green. The names of closely related strains to this study’s strains are shown in blue. Branches holding this study’s strain with very high bootstrap values (>50%) are shown in red to depict isolates with very close evolutionary distance.

Supplementary Table 1 Primers, PCR conditions, & PCR Results.

Supplementary Table 2 De-identified demographic data of the human hosts from which the strains were isolated.

Supplementary Table 3 Raw sequence and genomic data of the strains.

Supplementary Table 4 Phylogenomic and comparative genomic data of the strains.
