# Supplementary figures and images for: Molecular epidemiology of multidrug-resistant Klebsiella pneumoniae, Enterobacter cloacae, and Escherichia coli outbreak among neonates in Tembisa hospital, South Africa
Source: Front Cell Infect Microbiol. 2024 Feb 28;14:1328123. doi: 10.3389/fcimb.2024.1328123 (PMC10933102; doi:10.3389/fcimb.2024.1328123)

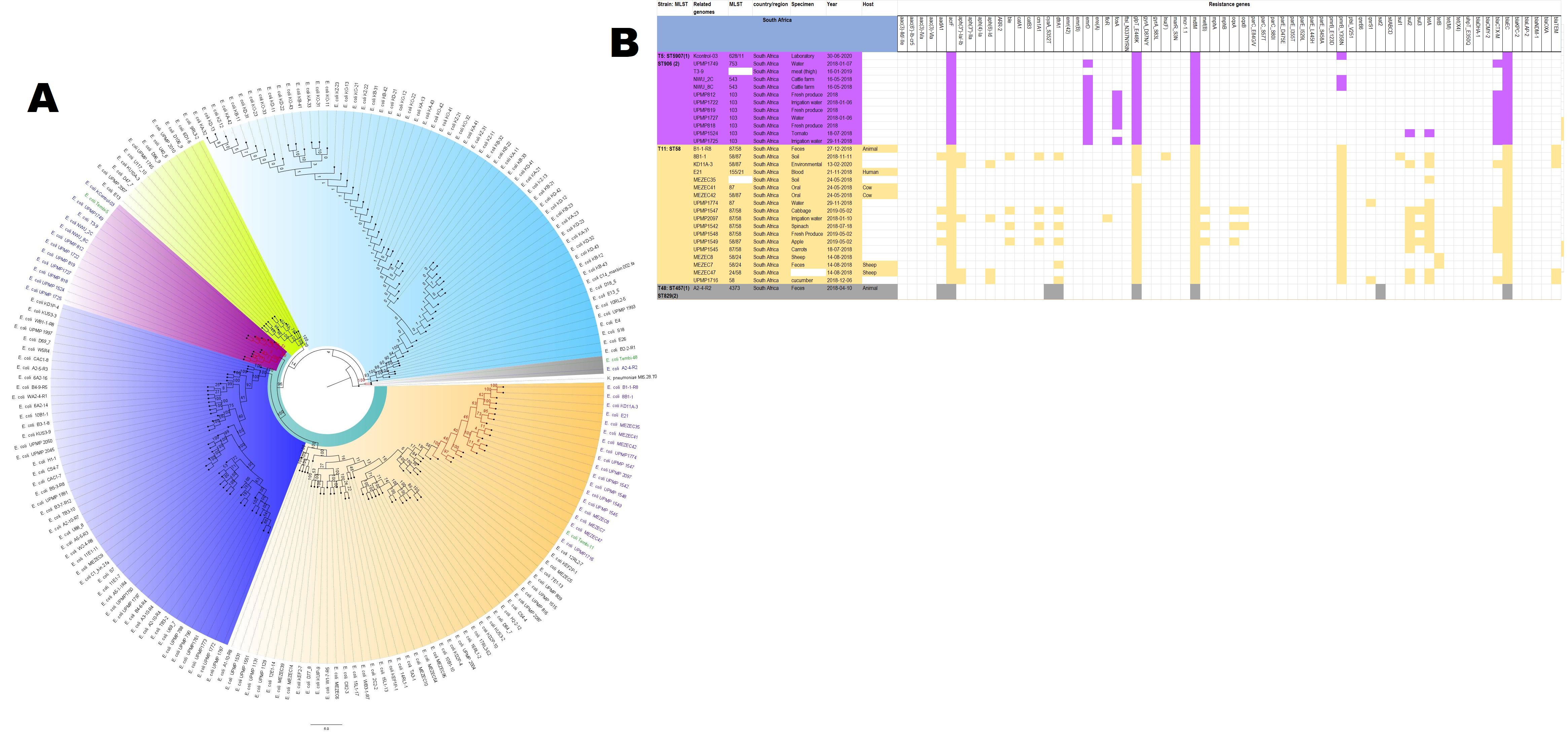

Supplement: Supplementary Figure 1 — Comparative phylogenomics of Escherichia coli strains from both this study and other strains from South Africa. The names of the strains from this study are colored green. The names of closely related strains to this study’s strains are shown in blue. Branches holding this study’s strain with very high bootstrap values (>50%) are shown in red to depict isolates with very close evolutionary distance. [file Image_1.tif]

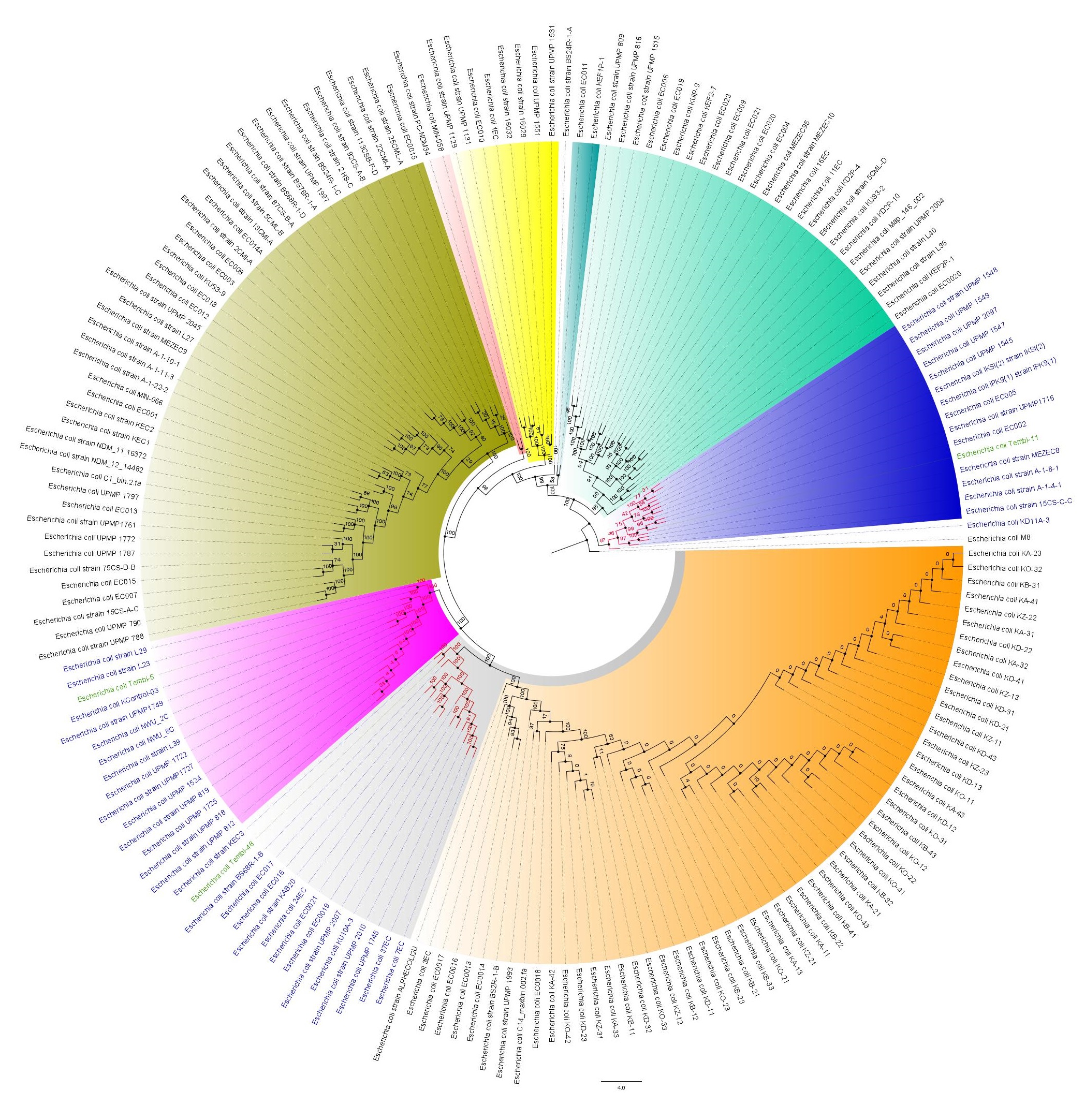

Supplement: Supplementary Figure 2 — Comparative phylogenomics of Escherichia coli strains from both this study and other strains from Africa. The names of the strains from this study are colored green. The names of closely related strains to this study’s strains are shown in blue. Branches holding this study’s strain with very high bootstrap values (>50%) are shown in red to depict isolates with very close evolutionary distance. [file Image_2.jpeg]

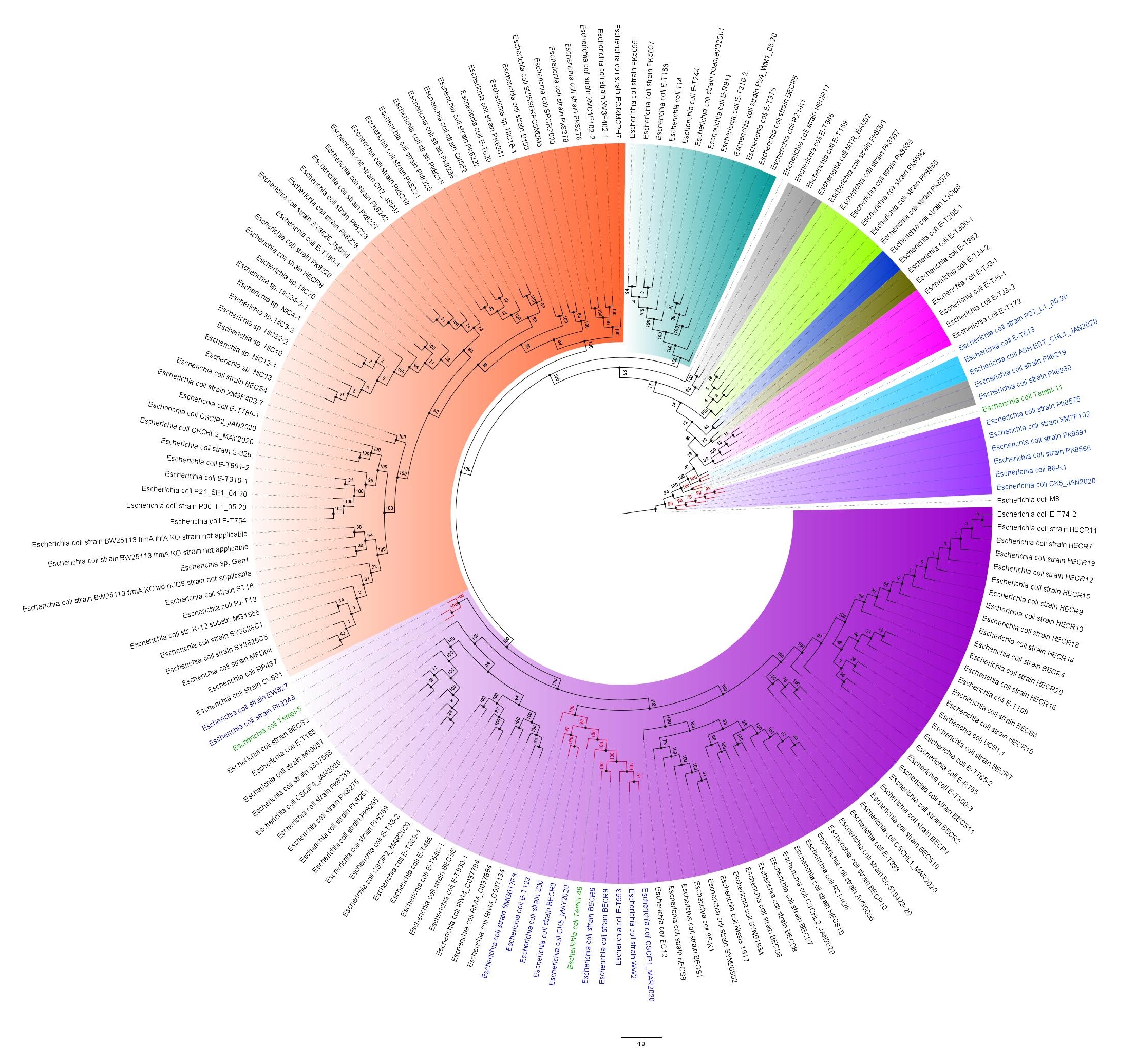

Supplement: Supplementary Figure 3 — Comparative phylogenomics of Escherichia coli strains from both this study and other strains from the world. The names of the strains from this study are colored green. The names of closely related strains to this study’s strains are shown in blue. Branches holding this study’s strain with very high bootstrap values (>50%) are shown in red to depict isolates with very close evolutionary distance. [file Image_3.jpeg]

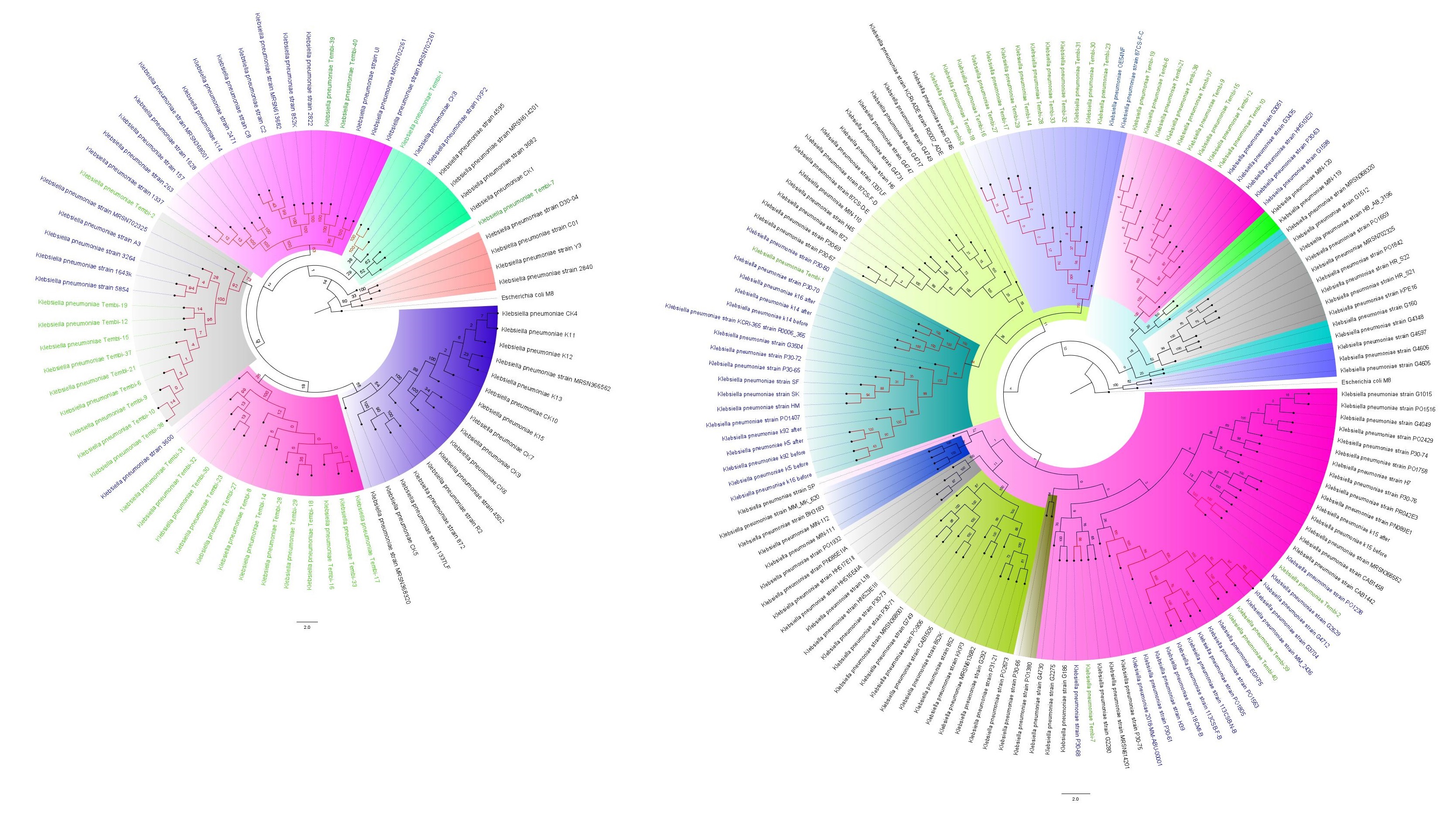

Supplement: Supplementary Figure 4 — Comparative phylogenomics of Klebsiella pneumoniae strains from both this study and other strains from Africa. The names of the strains from this study are colored green. The names of closely related strains to this study’s strains are shown in blue. Branches holding this study’s strain with very high bootstrap values (>50%) are shown in red to depict isolates with very close evolutionary distance. [file Image_4.jpeg]

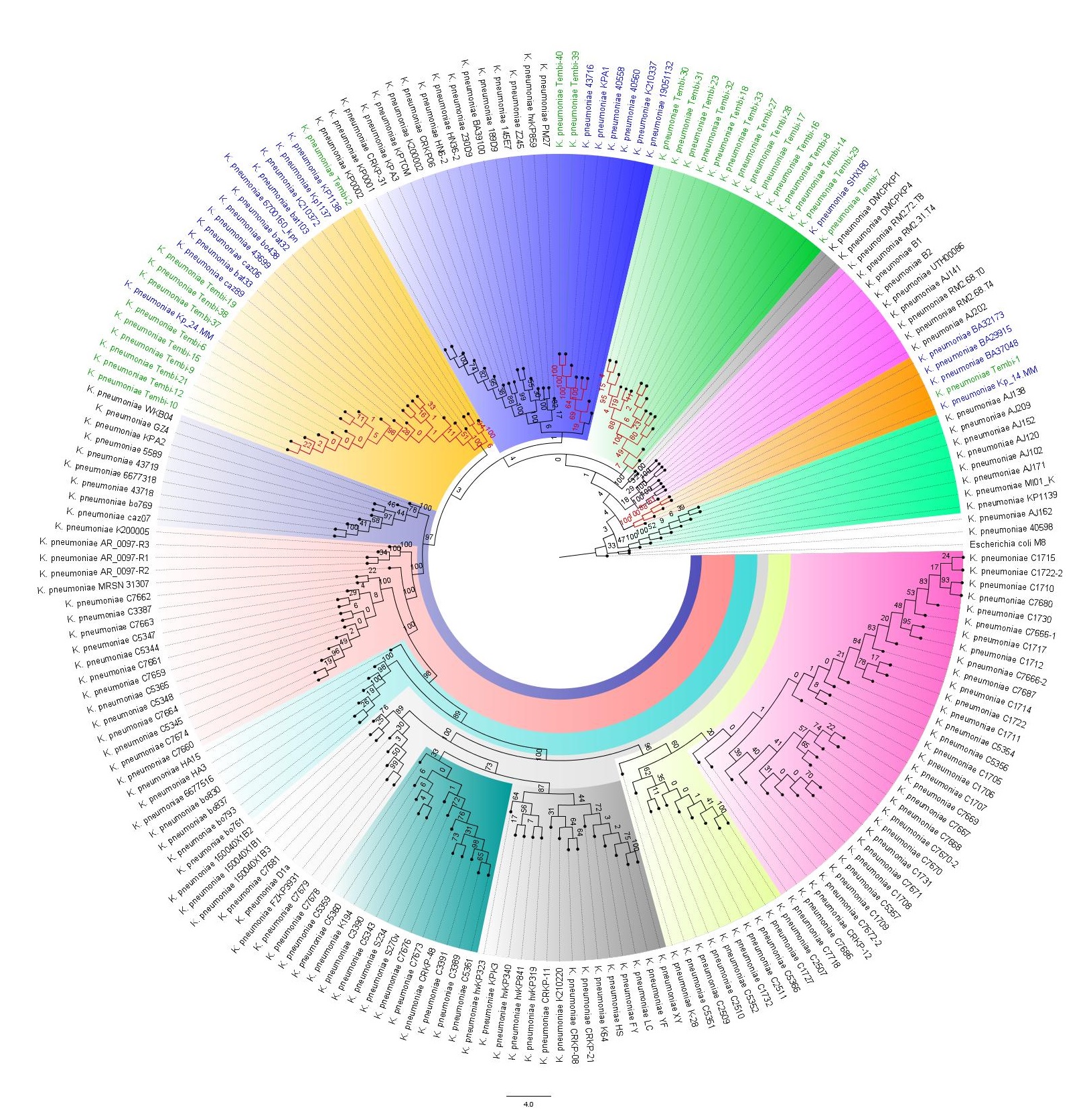

Supplement: Supplementary Figure 5 — Comparative phylogenomics of Klebsiella pneumoniae strains from both this study and other strains from the world. The names of the strains from this study are colored green. The names of closely related strains to this study’s strains are shown in blue. Branches holding this study’s strain with very high bootstrap values (>50%) are shown in red to depict isolates with very close evolutionary distance. Both S5A and S5B are phylogenetic analyses of global strains. [file Image_5.jpeg]

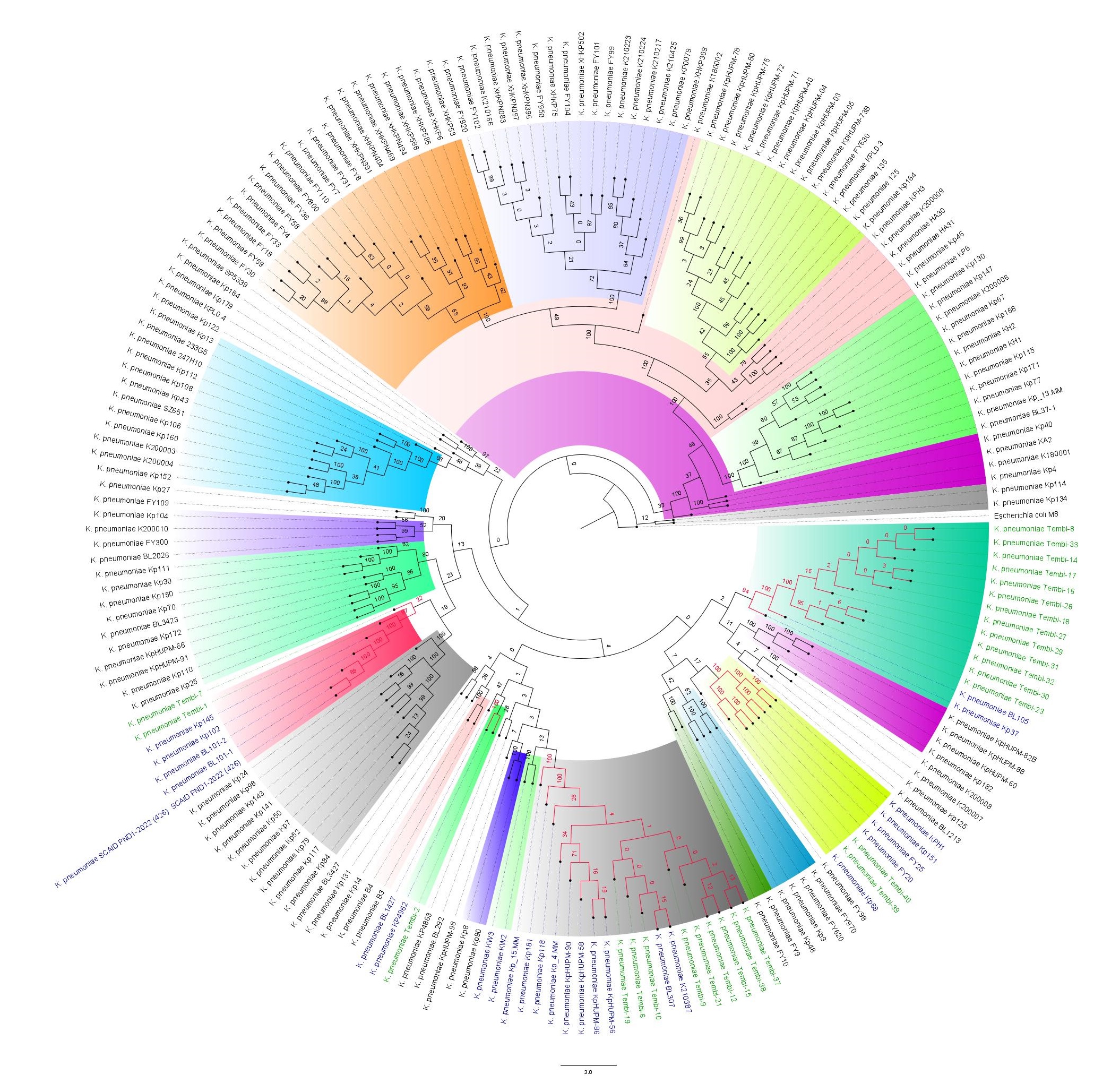

Supplement: Supplementary Figure 6 — Comparative phylogenomics of Enterobacter cloacae strains from both this study and other strains from Africa. The names of the strains from this study are colored green. The names of closely related strains to this study’s strains are shown in blue. Branches holding this study’s strain with very high bootstrap values (>50%) are shown in red to depict isolates with very close evolutionary distance. [file Image_6.jpeg]

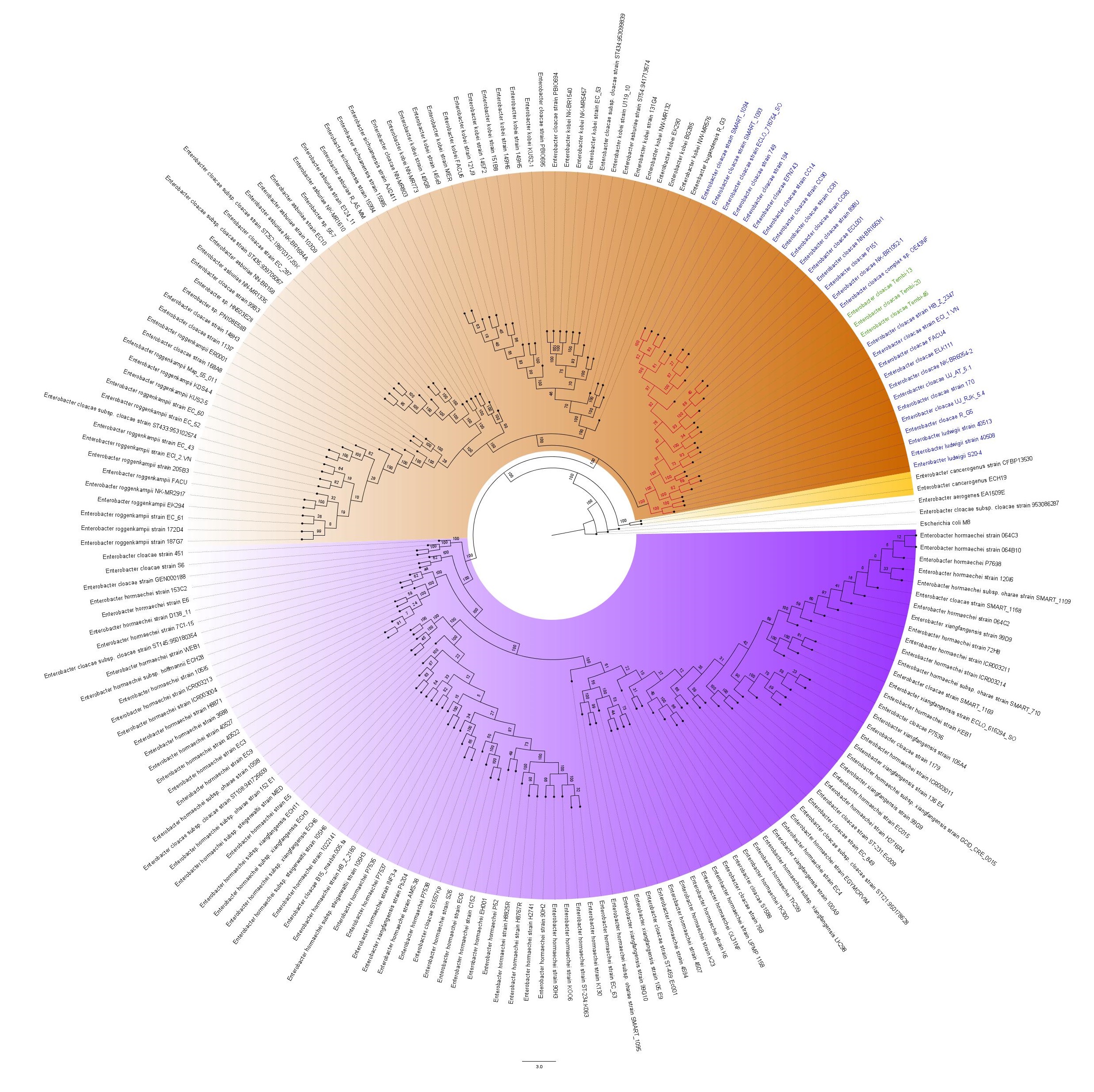

Supplement: Supplementary Figure 7 — Comparative phylogenomics of Citrobacter portucalensis strains from both this study and other strains from the whole world. The names of the strains from this study are colored green. The names of closely related strains to this study’s strains are shown in blue. Branches holding this study’s strain with very high bootstrap values (>50%) are shown in red to depict isolates with very close evolutionary distance. [file Image_7.jpeg]

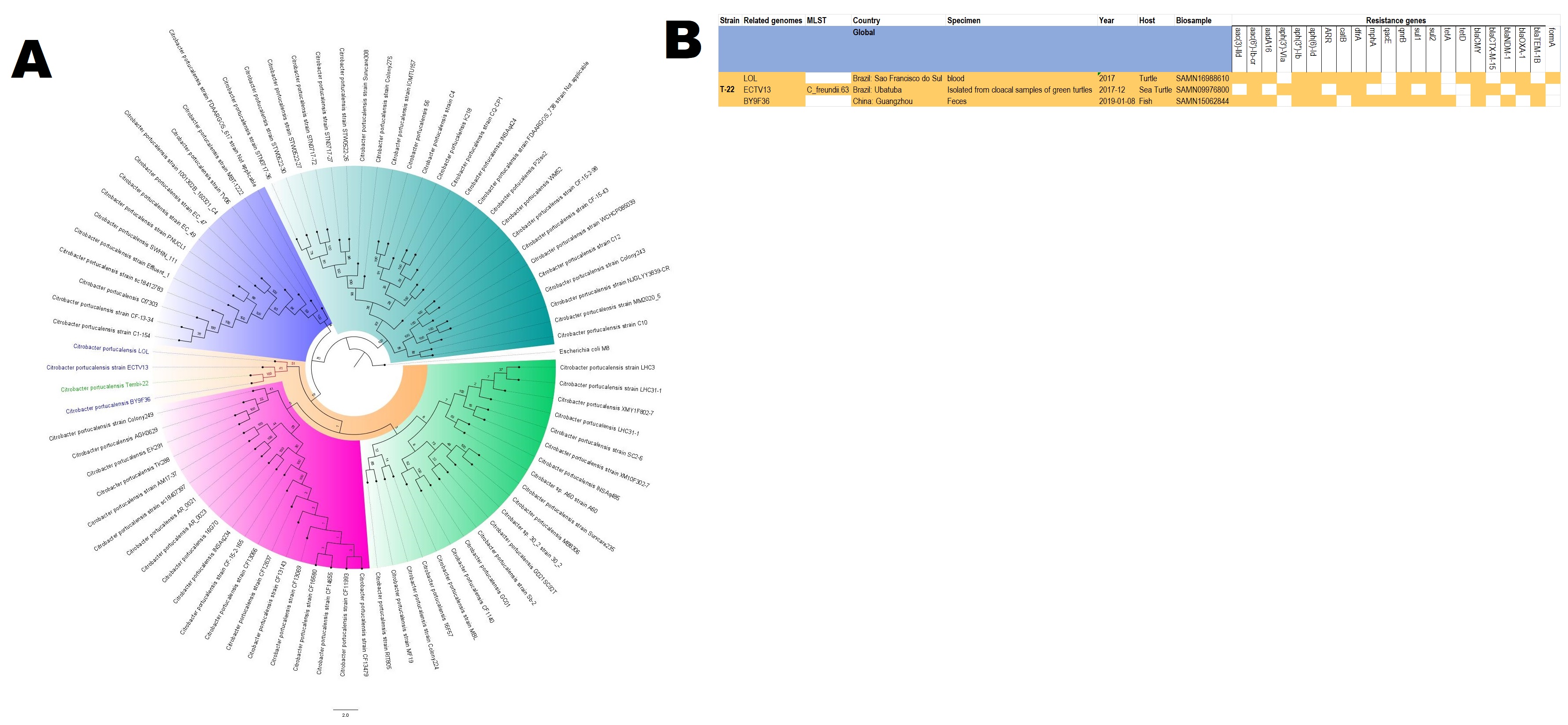

Supplement: Supplementary file 12 [file Image_8.jpeg]
